# Prognostic factors for survival in colorectal cancer patients with brain metastases undergoing whole brain radiotherapy: multicenter retrospective study

**DOI:** 10.1038/s41598-020-61354-y

**Published:** 2020-03-09

**Authors:** Taeryool Koo, Kyubo Kim, Hae Jin Park, Sae-Won Han, Tae-You Kim, Seung-Yong Jeong, Kyu Joo Park, Eui Kyu Chie

**Affiliations:** 1 Department of Radiation Oncology, Hallym University College of Medicine, Anyang, Republic of Korea; 2 Department of Radiation Oncology, Ewha Womans University College of Medicine, Seoul, Republic of Korea; 3 Department of Radiation Oncology, Hanyang University College of Medicine, Seoul, Republic of Korea; 4 Department of Internal Medicine, Seoul National University College of Medicine, Seoul, Republic of Korea; 5 Department of Surgery, Seoul National University College of Medicine, Seoul, Republic of Korea; 6 Department of Radiation Oncology, Seoul National University College of Medicine, Seoul, Republic of Korea

**Keywords:** Radiotherapy, Colorectal cancer, Metastasis

## Abstract

Whole brain radiotherapy (WBRT) is a mainstay of the treatment for brain metastases (BM). We evaluated prognostic factors in colorectal cancer (CRC) patients undergoing WBRT for BM. The medical records of 106 CRC patients undergoing WBRT for BM between 2000 and 2014 at three institutions were reviewed. Patient and tumor factors were analyzed to identify the prognostic factors for overall survival (OS) calculated from the date of BM diagnosis to the date of death or last follow-up. Surgical resection of BM was performed in six patients. The dose of WBRT was 30 Gy, and boost radiotherapy or stereotactic radiosurgery (8–23 Gy) was given to 15 patients. Systemic therapy for BM was administered in one patient before WBRT and 26 patients after WBRT. The median follow-up time was 3.9 months (range, 0.4–114.1 months). The median OS time was 3.9 months, and the 1-year OS rate was 18.2%. Older age (>65 years), multiple BM (≥3), elevated level of carcinoembryonic antigen (CEA, >5 ng/ml) at BM diagnosis, and extracranial metastases were adverse prognostic factors for OS. Patient with 0–1 factor showed better OS (at 1 year, 76.9%) than patients with 2 factors (16.7%) or 3–4 factors (4.2%; *p* < 0.001). In conclusion, we evaluated age, the number of BM, CEA level, and extracranial metastases as the prognostic factors for OS in CRC patients undergoing WBRT. Our result might be useful to develop prognostic models predicting survival for patients whom WBRT is intended for.

## Introduction

According to population-based studies, about 20% of patients with colorectal cancer (CRC) have distant-stage disease^1^. Liver and lung metastases are most frequent, found in about 20% to 30% of CRC patients, including those with synchronous and metachronous disease^2,3^. Brain metastasis (BM) rarely occurs in CRC patients, with an incidence of less than 3%^4–6^, but the prognosis is poor. The median survival time is less than 6 months after the diagnosis of BM^4,7^. Retrospective studies have reported young age, a single BM lesion, the absence of extracranial metastases, and a lower level of carcinoembryonic antigen (CEA) as good prognostic factors in CRC patients with BM^4,7,8^.

Validated prognostic tools that classify BM patients according to their predicted survival times can offer useful guidance for BM treatment. The Radiation Therapy Oncology Group (RTOG) performed recursive partitioning analysis (RPA) using a database from consecutive RTOG trials and suggested three classes based on performance status, age, primary tumor status, and extracranial metastases^9^. The Graded Prognostic Assessment (GPA) is the updated diagnosis-specific prognostic index, which includes additional prognostic factors such as number of BM and intrinsic subtypes of breast cancers^10^. CRC is not specified as a separate cancer subtype but is combined with other gastrointestinal (GI) cancers in the GPA indices. In this context, we previously presented a novel CRC-specific GPA index for patients with CRC who received any kind of BM treatment^11^. The novel index included number of BM, CEA level, and the presence or absence of neurologic symptoms^11^.

In terms of the treatment for BM, whole brain radiotherapy (WBRT) is a traditional and essential modality. In randomized clinical trials, more than 60% of BM patients treated with WBRT have shown complete or partial responses^12^. Although more aggressive local treatment modalities such as surgical resection or stereotactic radiosurgery (SRS) have been actively employed in recent decades for patients with good performance status and a limited number of BM, the addition of WBRT can reduce intracranial relapses and neurologic deaths^13^. However, prognostic factors in CRC patients undergoing WBRT for BM have not been evaluated in prospective trials as well as large retrospective studies. In this study, we aim to evaluate prognostic factors which could attribute to develop prognostic tools in CRC patients undergoing WBRT for BM.

## Results

Participating institutions collected 113 patients who received WBRT for BM from CRC. We excluded five patients who were lost to follow-up, one patient who underwent WBRT for pachymeningeal metastasis, and one patient with insufficient information of BM. Finally, 106 patients were included in this study. When the primary tumor was diagnosed, 10 patients (9.4%) had BM and 33 patients (31.1%) had systemic disease other than BM. The median time interval between the diagnosis of primary tumor and BM was 22 months (range, 0–132 months). The median age at the time of BM diagnosis was 62 years (range, 31–85). Regarding the number of BM, 41 patients (38.7%) had a single BM, 21 (19.8%) had two lesions, 14 (13.2%) had three lesions, and 30 (28.3%) had four or more lesions. The median CEA level at the time of BM diagnosis was 29.75 ng/dL (range, 0.5–7,620 ng/dL). Neurologic symptoms were noted in 71 patients (67.0%): motor weakness (*n* = 45), gait disturbance (*n* = 17), seizure (*n* = 17), dysarthria (*n* = 14), disorientation (*n* = 10), and other symptoms (*n* = 8). Extracranial metastases were found in 86 patients (81.1%): the lungs (*n* = 68), lymph nodes (*n* = 47), liver (*n* = 44), bones (*n* = 31), and other locations (*n* = 26). Details of patient and tumor characteristics are summarized in Table 1.

**Table 1:** Patient characteristics of the patients with brain metastases from colorectal cancers.

Variables		N	(%)
Median age (years)		62	(range, 31–85)
Sex	Male	64	(60.4)
	Female	42	(39.6)
Primary sites	Colon	54	(50.9)
	Rectum	52	(49.1)
Primary laterality	Right	20	(18.9)
	Left	86	(81.1)
ECOG performance	1	31	(29.2)
	2	40	(37.7)
	3	26	(24.5)
	4	9	(8.5)
Neurologic symptoms	Yes	71	(67.0)
	No	35	(33.0)
Location of BM	Supratentorial	51	(48.1)
	Infratentorial	16	(15.1)
	Both	39	(36.8)
Number of BM	1	41	(38.7)
	2	21	(19.8)
	3	14	(13.2)
	4	12	(11.3)
	5	1	(0.9)
	≥6	17	(16.0)
Accompanied hemorrahge	Yes	23	(21.7)
	No	83	(78.3)
Primary control	Controlled	86	(81.1)
	Uncontrolled	20	(18.9)
Extracranial metastases	Yes	86	(81.1)
	No	20	(18.9)
CEA*	≤5 ng/ml	24	(22.6)
	>5 ng/ml	74	(69.8)

ECOG Eastern Cooperative Oncology Group, BM brain metastasis, CEA carcinoembryonic antigen.

*Missing data were included.

Surgical resection of BM was performed in six patients (5.7%). The dose of WBRT was 30 Gy. Of the patients, 13 received boost radiotherapy (8 Gy to 20 Gy), and two patients underwent SRS (18 Gy and 23 Gy). One patient received systemic therapy, regorafenib, for BM before WBRT. After WBRT, systemic chemotherapy was administered in 26 patients: 11 patients received 5-fluorouracil (5-FU)- and oxaliplatin-based regimens, while the following three approaches were used in five patients each: 5-FU- and irinotecan-based, capecitabine-based, and other regimens.

**Figure 1:**
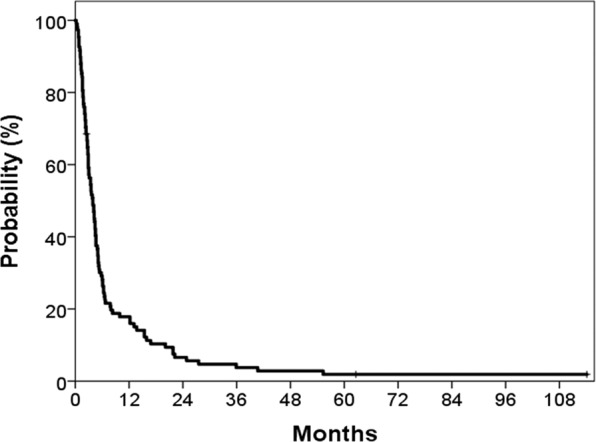
Survival curves of patients undergoing whole brain radiotherapy for brain metastases from colorectal cancer.

The median follow-up time was 3.9 months (range, 0.4–114.1 months). Overall, 103 patients (97.2%) died, two patients were alive, and one patient was lost to follow-up. The median overall survival (OS) time was 3.9 months, and OS rates were 28.7% at 6 months and 18.2% at 1 year (Fig. 1). Follow-up imaging studies were performed in 39 patients after WBRT. Complete response was observed in 4 patients, partial response in 9, stable disease in 11, and progressive disease in 15. Regarding salvage treatments, SRS (median 18 Gy; range, 15–22 Gy) was administered in seven patients, surgical resection and SRS (17 Gy and 18 Gy) in two patients, and WBRT (20 Gy) in another three patients.

**Figure 2:**
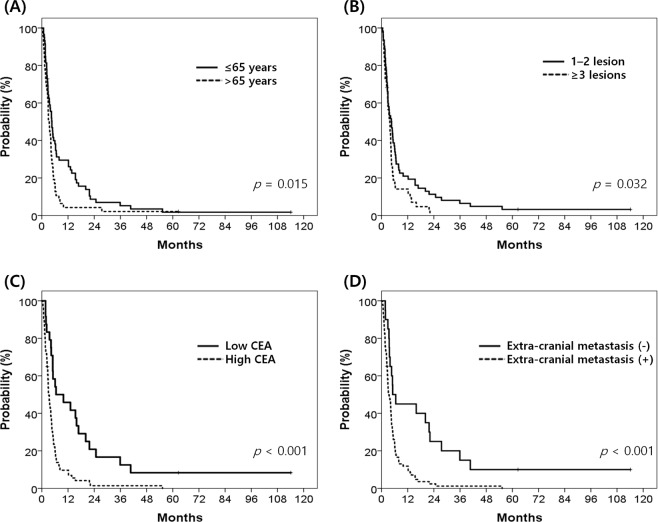
Survival curves according to the prognostic factors for overall survival: (**A**) age, (**B**) the number of brain metastases, (**C**) the level of carcinoembryonic antigen at brain metastases, and (**D**) presence of extracranial metastases.

Table 2 lists the results of the univariate analyses. Age, presence of neurologic symptoms, number of BM, presence of extracranial metastases, and level of CEA showed statistical significances. After adjusting for covariates, age, number of BM, presence of extracranial metastases, and level of CEA were confirmed as prognostic factors (Table 2). Patients with age > 65 years, BM ≥ 3, CEA level > 5 ng/mL, or extracranial metastases had lower OS (Fig. 2).

**Table 2:** Univariate and multivariate analyses of prognostic factors for overall survival.

Variables		N	Univariate	p	Multivariate	p
			1-year OS (months)		HR (95% CI)	
Age	≤65	59	29.5	0.015	Reference	0.006
	>65	47	4.3		1.850 (1.192–2.873)	
Sex	Male	64	15.6	0.055		
	Female	42	22.1			
Primary sites	Colon	54	15.2	0.217		
	Rectum	52	21.2			
Primary laterality	Right	20	15.0	0.203		
	Left	86	18.9			
ECOG	1	31	32.3	0.086		
	2–4	75	12.3			
Neurologic symptoms	No	35	26.7	0.016		NS
	Yes	71	14.1			
Location of BM	Supratentorial	51	15.7	0.219		
	Infratentorial	16	31.3			
	Both	39	16.0			
Number of BM	1–2	62	21.0	0.032	Reference	0.039
	≥3	44	14.1		1.575 (1.024–2.422)	
Hemorrhage	No	83	18.4	0.828		
	Yes	23	17.4			
Primary control	Controlled	86	16.3	0.976		
	Uncontrolled	20	26.7			
Extracranial metastases	No	20	45.0	<0.001	Reference	0.007
	Yes	86	11.8		2.549 (1.298–5.006)	
CEA	≤5	24	45.8	<0.001	Reference	0.013
	>5	74	9.7		1.956 (1.155–3.312)	

*OS* overall survival, *HR* hazard ratio, *CI* confidence interval, *ECOG* Eastern Cooperative Oncology Group, *NS* not significant, *BM* brain metastasis, *CEA* carcinoembryonic antigen.

**Figure 3:**
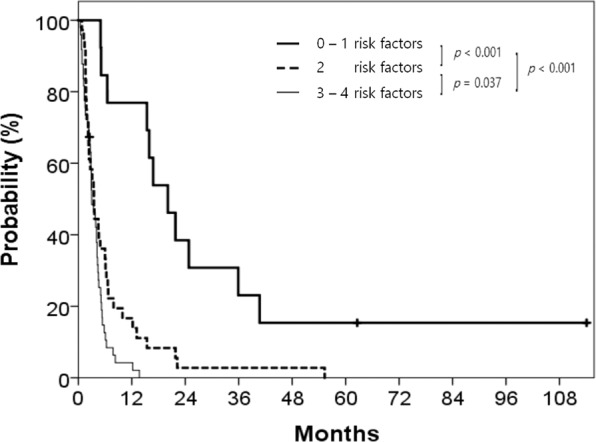
Survival curves according to the number of risk factors.

We classified patients as 3 risk groups according to the prognostic factors: high-risk (3–4 factors; *n* = 13), intermediate-risk (2 factors; *n* = 36), and low-risk (0–1 factor; *n* = 49) groups. The patients who had missing data of CEA level were excluded. The risk groups showed statistically significant differences in OS (Fig. 3). At 1 year, OS rates were 76.9% for low-risk group, 16.7% for intermediate-risk group, and 4.2% for high-risk group (*p* < 0.001).

## Discussion

With the improvement in survival outcomes and imaging studies, the incidence of BM is increasing in patients with cancer. WBRT has been the most common treatment modality for BM. Although the benefit of SRS for patients with a limited number of BM has been emphasized^14,15^, WBRT still has an important role in the treatment of patients unsuitable for SRS or surgery, in the management of multiple BM^16^, and as adjuvant treatment after SRS or surgery^13,17^.

Traditionally, the homogeneous distribution of radiation dose to the entire brain has been the preferred WBRT plan. The hippocampi are associated with the formation and recall of new memories, and radiation to the hippocampus induces neural precursor cell dysfunction by the ablation of hippocampal neurogenesis^18^. Even a relatively modest radiation dose to the bilateral hippocampi can induce neurocognitive function impairment^19^. To decrease the dose of radiation to the hippocampal area, hippocampal-avoidance WBRT (HA-WBRT) was introduced. The RTOG performed a phase II trial (RTOG 0933) examining memory preservation using the HA-WBRT technique. Compared with historical data, patients undergoing HA-WBRT had significantly lower memory decline^20^.

Despite the dosimetric benefit of HA-WBRT, its high cost remains the biggest hurdle working against the wide use of this technique. In Korea, the medical cost was more than $4,000 U.S. for 10 fractions of HA-WBRT, but less than $1,000 U.S. for 10 fractions of conventional WBRT. Administration of HA-WBRT could be justified in patients with longer expected survival because the technique can mitigate neurocognitive dysfunction after WBRT. In patients with short life expectancy, however, conventional WBRT may have a better rationale considering its shorter planning time and lower cost^21^.

Within the context of the cost-effectiveness of WBRT techniques, prognosis prediction becomes even more important. By predicting the prognosis of these patients, we could then elaborate WBRT planning and intensify systemic treatments, or save excessive medical costs and concentrate on supportive care. However, due to the rareness of disease condition, even prognostic factors in CRC patients undergoing WBRT for BM have not been clearly evaluated.

Actually, previously reported prognostic indices, such as the RPA and GPA, are not specific for primary sites^9,22^. Although the GPA was refined to be diagnosis-specific, CRC was not appreciated as a separate entity but instead included with GI cancers, where the only prognostic factor is Karnofsky Performance Score (KPS)^10,23^. In fact, when applied to the current cohort, the RPA, GPA, and diagnosis-specific GPA were incapable of patient stratification, with *p*-values exceeding 0.05 (data not shown).

In this study, we revealed four prognostic factors for OS in patients with BM from CRC: age, level of CEA at BM, presence of extracranial metastases, and number of BM. Our results would be useful to develop prognostic models predicting survival for patients whom WBRT is intended for.

Patients’ performance status is the weighted prognostic factor in the RPA and GPA, but not in our study. Differences between KPS’s and Eastern Cooperative Oncology Group (ECOG)’s scoring systems could be a source of potential bias. We used the ECOG grading system, but the RPA and GPA used the KPS, which has a more subdivided scale. A KPS of 70 to 90 could be regarded as grade 1 under the ECOG Performance Status, but the GPA system scores different points for a KPS of 70, 80, or 90. The subjective aspect of performance evaluation might be another source of bias because data was retrospectively reviewed by investigators from different institutions. Although performance status has not always been reported as a prognostic factor in other studies of BM from CRC^4,8,24–30^, a prospective study needs to confirm its prognostic value.

The number of BM is another well-known prognostic factor for patients with BM. This factor, as in fewer than four BM, is incorporated in the diagnosis-specific GPA for lung cancer, melanoma, and renal cell carcinoma^10,23^. Practically speaking, the number of BM affects choice of treatment. For patients with a limited number of BM, SRS is preferred over WBRT alone^14,15,17^. The prognostic value of the number of BM has also been reported in previous studies of BM from CRC, though the studies were retrospectively designed because of the rareness of the situation^30^. In this context, we tried to perform log-rank tests for each BM number and found statistical significance with a cut-off of three lesions (Table 2).

The fact that patients with a limited number of BM received WBRT rather than SRS in our study could be a point of discussion. The volume effect, which we did not examine, might be an underlying cause. Like BM number, larger cumulative BM volume is regarded as a poor prognostic factor^31^. We could presume that our patients with a limited BM number might have had a relatively large BM volume because only eight patients underwent surgical resection or SRS rather than WBRT alone. It would be clinically meaningful if a prognostic index encompassing cumulative BM volume were investigated.

CEA level is one of the most useful tumor markers for CRC. An elevation means a larger entire tumor burden and higher probability of metastatic disease, as CEA-related cell adhesion molecules participate in tumor progression, angiogenesis, and metastasis^32^. CEA level can be used to estimate prognosis and monitor recurrence after complete CRC resection^33^. The association of CEA level with survival has also been confirmed in patients with metastatic CRC^34,35^. Particularly in patients with BM from CRC, a higher level of CEA was associated with lower survival rates^8,29^.

As expected, an elevated level of CEA at the time of BM diagnosis was associated with lower OS in this study. CEA elevation may be a surrogate for systemic tumor burden. Thus, the significance of extracranial metastases was tested, and, as expected, these metastases were found to be prognostic. Extracranial metastases were more commonly found in patients with an elevated CEA level than in patients with a CEA level within normal range (94.6% vs. 54.2%; *p* < 0.001). Despite the correlation, both elevated CEA level and presence of extracranial metastases independently showed prognostic significance in the multivariate analysis.

Based on four prognostic factors, we classified the patients as 3-tier risk groups and confirmed significant differences in OS. Our results are needed to be validated in prospective trials or large population studies. Another limitation of this study was the insufficient imaging information before and after WBRT for the evaluation of treatment response. This limitation represents an inherent restriction of retrospective, multicenter studies conducted for a rare disease.

In conclusion, age, level of CEA at BM, presence of extracranial metastases, and number of BM were evaluated to be prognostic factors for OS in CRC patients undergoing WBRT for BM. The prognostic factors might be helpful to develop prognostic models selecting patients who are expected to have a good prognosis. Further validation using larger independent cohorts is needed.

## Methods

Three Korean institutions participated in this study after receiving approval from the institutional review board of Seoul National University Hospital, Ewha Womans University Mokdong Hospital, and Hallym University Sacred Heart Hospital. All procedures performed in studies were in accordance with the ethical standards of the institutional review board of each hospital and with the 1964 Helsinki declaration and its later amendments or comparable ethical standards. Also, a waiver of informed consent was approved by each institutional review board.

Included in the study were CRC patients who underwent WBRT for BM diagnosed by imaging studies between 2000 and 2014. Magnetic resonance imaging (MRI) or computerized tomography (CT) was used for the imaging diagnosis. Patients with multiple primary cancers were excluded. The collected data included patient characteristics, clinicopathological information about primary tumors and BM lesions, and details of treatment for BM. Regarding the location of the primary tumor, ascending and transverse colon cancers were classified as right CRC, and descending colon, sigmoid colon, and rectal cancers as left CRC. Patient performance at BM diagnosis was graded using the ECOG performance scale. Motor weakness, gait disturbance, disorientation, dysarthria, visual disturbance, consciousness change, and seizure were recorded as neurologic symptoms^11^.

Statistical analyses were performed using Predictive Analytics Software, version 18.0 (SAP America Inc., Newtown Square, PA, USA). We calculated OS from the date of BM diagnosis to the date of death or last follow-up. The Kaplan–Meier method was used to estimate the OS rates and plot survival curves. Univariate and multivariate analyses were performed using the log-rank test and Cox proportional hazards model, respectively. The multivariate analyses included variables that showed a *p* value < 0.05 in the univariate analyses.

## Data Availability

The datasets generated during and/or analysed during the current study are not publicly available due to personal information protection act in Republic of Korea.
